# Feasibility evaluation of the Versius surgical system: robot-assisted hysterectomy for benign and malignant gynaecological lesions

**DOI:** 10.1007/s00404-024-07655-3

**Published:** 2024-07-24

**Authors:** Pawel Sadlecki, Malgorzata Walentowicz-Sadlecka

**Affiliations:** 1 Medical Department, University of Science and Technology, Bydgoszcz, Poland; 2 Department of Obstetrics, Gynaecology and Gynaecologic Oncology, Regional Polyclinical Hospital, Grudziadz, Poland

**Keywords:** Minimally invasive surgery, Robotic assisted hysterectomy, Robotic assisted radical hysterectomy, CMR Versius

## Abstract

**Introduction:**

The application of minimally invasive surgery allows for radical and precise removal of the gynaecological lesion while simultaneously reducing the side effects and complications associated with surgical treatment. This paper aims to share our direct experience with the implementation of the CMR Versius robotic platform in the treatment of benign and malignant gynaecological lesions.

**Methods:**

This study included patients who underwent hysterectomy in the Department of Obstetrics, Gynaecology, and Gynaecologic Oncology at the Regional Polyclinical Hospital in Grudziadz, Poland. A total of 50 patients were included in the study: 29 underwent laparoscopic surgery and 21 underwent robot-assisted surgery using the CMR Versius system.

**Results:**

It was found that in the case of non-radical hysterectomy, the duration of surgery differed significantly (96.5 vs. 134.6 min, *p* < 0.01) in the groups of patients undergoing laparoscopic and robotic surgery. There were also no statistically significant differences in loss of blood parameters, rate of complications and conversions to other type of surgery after the laparoscopic and robotic surgeries. Both groups did not differ significantly in terms of hospitalisation time after surgery.

**Conclusion:**

Versius CMR surgical robot assistance provides safe and effective support for MIS procedures in gynaecology.

## Introduction

Hysterectomy, a surgical procedure commonly performed by gynaecologists, is utilised to treat various non-cancerous and cancerous conditions affecting the uterus. The emergence of endoscopic surgery has marked a significant milestone in this field, with the first laparoscopic hysterectomy performed by Harry Reich in Kingston, Pennsylvania, in 1988, followed by the introduction of robotic hysterectomy by Concepcion Diaz-Arrastia in 2002 [1]. Advancements in anaesthesia, blood transfusion, antibiotics, and surgical techniques have further contributed to the widespread adoption of this procedure. The most frequent indications for hysterectomy include pre-cancerous conditions of the cervix and endometrium, benign uterine tumours, unresponsive uterine bleeding, and malignant tumours of the cervix, uterus, and ovaries. The management of gynaecological cancers usually involves a combination of surgery, radiation, and chemotherapy treatments, with the goal of achieving the best possible clinical outcomes while minimising morbidity and preserving quality of life. Detecting cancer at an early stage of advancement or in a pre-cancerous state enables the utilisation of minimally invasive surgical techniques (MIS). The application of MIS in the treatment of gynaecological tumours allows for radical and precise removal of the lesion while simultaneously reducing the side effects and complications associated with surgical treatment [2].

In recent years, robot-assisted surgery has emerged as a promising approach for the treatment of gynaecological cancers. Robot-assisted minimally invasive surgery has effectively addressed several challenges associated with traditional approaches. These challenges include a limited range of surgical movement and unfavourable ergonomics for both the surgeon and bedside assistant [3]. Moreover, surgeon competency typically requires extensive training and involves a steep learning curve. However, the advent of robot-assisted techniques has significantly mitigated these issues. Such innovative approaches offer a stable, magnified three-dimensional view, effectively filters tremors, and enables motion scaling [4]. In addition, the use of articulated or wristed instruments provides greater degrees of movement, thereby facilitating precise tissue dissection and suturing. Overall, robot-assisted minimally invasive surgery has made remarkable progress in overcoming the aforementioned challenges and revolutionising the field of surgical procedures [5].

Versius, which was developed by CMR surgical in Cambridge, UK, is an innovative robotic surgical system designed to support surgeons in performing MIS. While the da Vinci surgical system is the most frequently used robotic platform in gynaecologic surgery, the newer CMR Versius surgical robotic system has been introduced as a viable alternative. The vast majority of the literature on the use of surgical robots in gynaecology is based on experiences resulting from the use of the da Vinci system. The main features that differentiate the da Vinci robot from the Versius system from CMR surgical include the console design, the 3D visualisation method, the system’s mobility, the design of the robot arms, and the operator’s position behind the robot console during surgery [6]. Versius has been meticulously crafted based on feedback from surgeons and their teams, with the goal of enhancing both the end-user experience and surgical outcomes. One notable feature of the system is its ability to replicate the articulation of the human arm, for which it relies on a wristed instrument tip that provides seven degrees of freedom within the patient’s body [7].

This paper aims to share our direct experience with the implementation of the CMR Versius robotic platform in the treatment of malignant and benign gynaecological lesions. Specifically, we compared hysterectomy procedures performed laparoscopically with the assistance of the Versius CMR surgical robot.

## Materials and methods

This study included patients who underwent surgery in the Department of Obstetrics, Gynaecology, and Gynaecologic Oncology at the Regional Polyclinical Hospital in Grudziadz, Poland, between June 2023 and December 2023, for both oncological and non-oncological conditions. A retrospective analysis was conducted using anonymous data pertaining to patient interviews, physical examinations, additional tests, indications, surgical treatment methods, and related parameters. The surgical procedures were performed by two specialists in obstetrics, gynaecology, and gynaecologic oncology, who were accredited, practising and high-volume consultant gynaecology surgeons with extensive experience in both open and minimally invasive surgery. The operators participating in the study had no prior experience with any available surgical robotic systems before undergoing training on the Versius CMR surgical robot. The surgeons and their assistants were properly trained by the company and had obtained a proficiency certificate based on simulation, dry lab, and cadaver lab experience. All procedures were performed under the supervision of the company team, which was composed of engineers, as part of an implementation programme.

### Patients

A total of 50 patients were included in the study: 29 underwent laparoscopic surgery, and 21 received robot-assisted surgery using the CMR Versius system. Patients were qualified for surgical treatment and the type of surgery (laparoscopy vs. robotic) by one operator. Qualifications for the type of surgery took place on an outpatient basis approximately 4 weeks before the planned surgery. The type of minimally invasive laparoscopic or robotic treatment was mainly dictated by the availability of the Versius robotic system in the hospital. Patients were qualified for surgical treatment due to the presence of benign lesions, such as cervical dysplasia, abnormal uterine bleeding, myoma, endometrial hyperplasia with atypia or adenomyosis (*N* = 28), or malignant lesions, such as endometrial cancer (*N* = 22). Before qualifying for treatment, all patients underwent cytology, gynaecological examination, ultrasound examination, and histopathological diagnosis of the endometrium. In some cases (*N* = 8), a diagnostic biopsy of the cervix was also performed after a colposcopy. Before surgical treatment, all endometrial cancer patients underwent imaging and histopathological tests confirming their qualification for treatment with minimally invasive techniques due to the low risk of disease recurrence and the early stage of clinical advancement of the disease. All patients underwent an anaesthesiologic consultation before surgical treatment.

### Procedures

Total hysterectomy was performed in 33 patients, 19 of which consisted of total laparoscopic hysterectomy (TLH), including 16 with bilateral salpingo-oophorectomy (BSO) and 3 with removal of the fallopian tubes (BS). In the other 14 procedures, robot assistance (TRH) was provided, including 11 with BSO and 3 with BS. A total of 17 patients underwent radical hysterectomy (mostly type A according to Querleu and Morrow), including 10 treated laparoscopically (RLH), and seven subject to robot-assisted (RRH) procedures. In this group, all patients underwent node biopsy or pelvic lymphadenectomy performed laparoscopically for staging purposes. All these patients were classified as low risk, with no need for systemic lymphadenectomy. The pathological specimens from the evaluated lymph node samples in all patients were free from neoplastic disease. The histopathological examination of the resected specimens, confirmed the correctness of the preoperative qualification and the undertaken procedure.

**Fig. 1:**
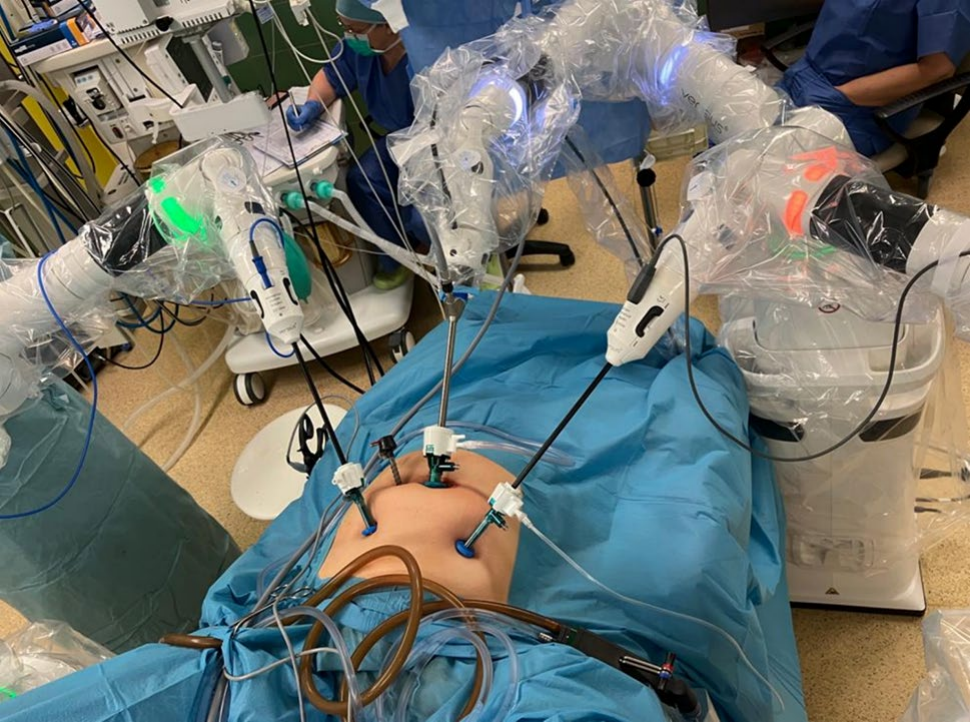
Bedside units (BSUs) localisation for robot-assisted hysterectomy. The following instruments were inserted through the trocars: left lower abdomen: bipolar Maryland grasper, right: monopolar scissors, right upper abdomen: assistant’s port (usually used to insert bipolar coagulation or suction), umbilicus: used to insert a camera

Port and bedside unit (BSU) placements were based on laparoscopic experience and technical advice from the company. Minor variations in the BSU positions were introduced during the procedures due to the conflict of the robotic arms, which is known as ‘clashing’ (Fig. 1).

Laparoscopic hysterectomy was performed as follows: after disinfection of the operating field with a Veress needle, pneumoperitoneum was performed, and CO2 operating pressures were typically 12 mmHg. Then, after making an incision in the umbilicus, a 10 mm trocar was inserted, through which a camera with 0° optics was placed. Then, the patient was placed in the Trendelenburg position at approximately 15°, and trocars were inserted under visual control. For the laparoscopic hysterectomy, the primary 10 mm port was placed at the umbilicus, followed by the attachment of three, 5 mm, ancillary trocars, one suprapubic and two laterally, to the epigastric arteries, in the left and right lower abdominal quadrants, respectively. In the case of a modified laparoscopic radical hysterectomy with lymph node removal, the suprapubic 5 mm trocar was replaced by a 10 mm trocar, through which the removed lymph nodes were retrieved in endo-bags. Laparoscopic hysterectomy was performed using bipolar forceps, disposable scissors, a suction device, and two graspers. Before starting the procedure, a SecuFix manipulator (Richard Wolf) was inserted into the uterus. The vagina was closed with two layers of V-loc 2.0 suture using laparoscopic needle drivers.

**Fig. 2:**
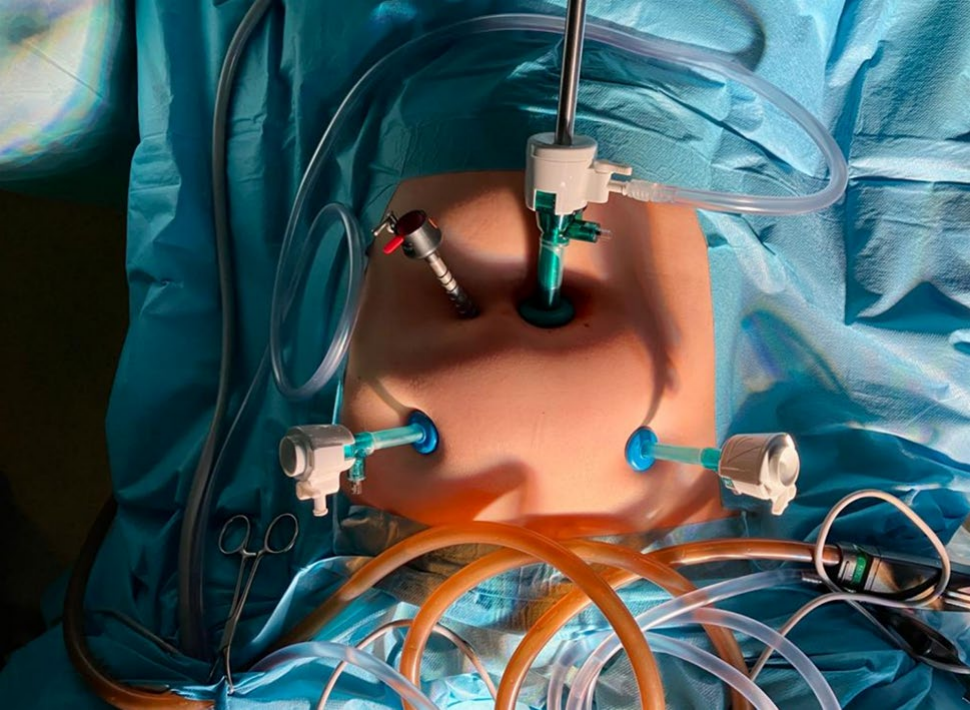
Trocars placement for non-radical robot-assisted hysterectomy

**Fig. 3:**
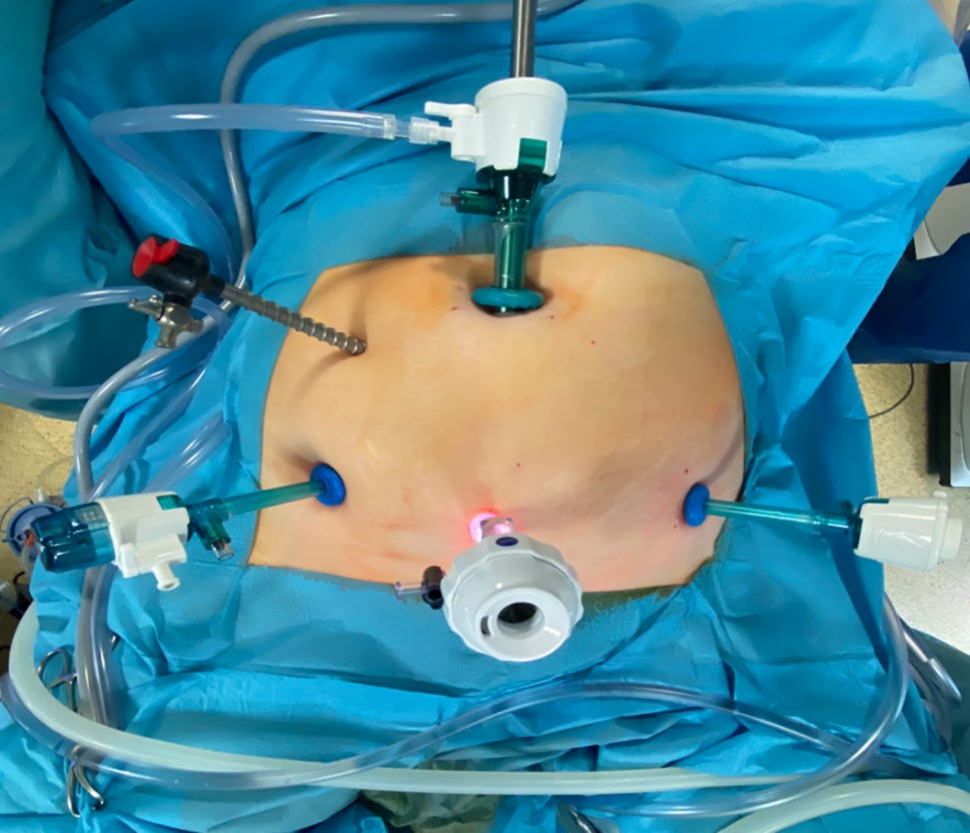
Trocars placement for radical robot-assisted hysterectomy

**Fig. 4:**
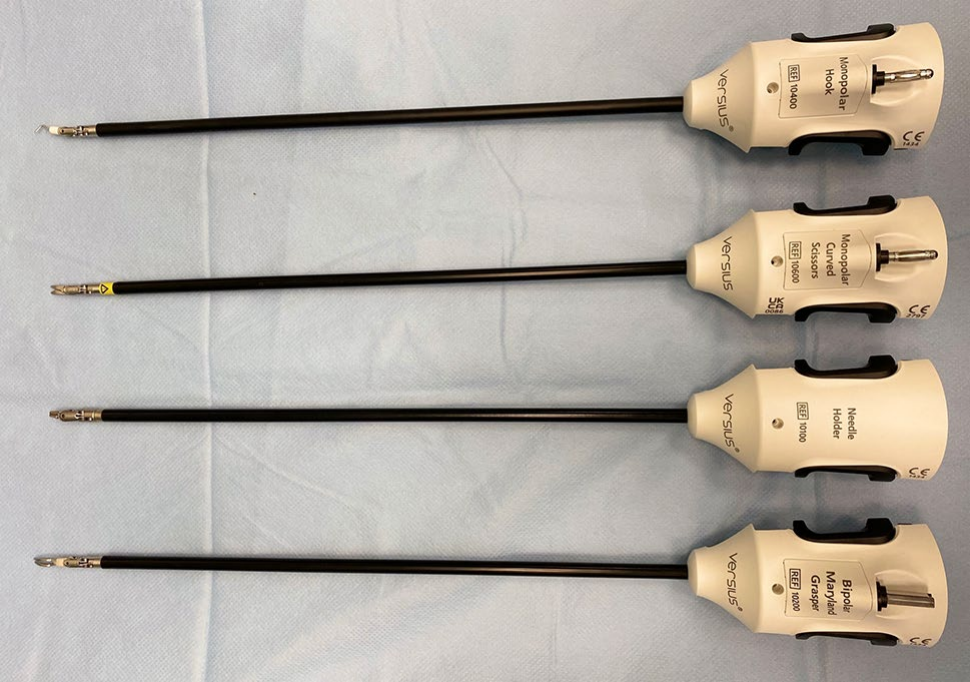
Robotic tools (from the top): monopolar hook, monopolar curved scissors, needle holder and bipolar Maryland grasper

During robotic surgery, patients were usually placed on a vacuum mattress to stabilise their position. In addition, shoulder supports and belts were used for both the laparoscopic and robotic surgeries. Robotic hysterectomy was performed as follows: after disinfection of the operating field with a Veress needle, pneumoperitoneum was usually performed at a pressure of 12 mmHg. The next step after making the incision in the umbilicus was the insertion of a 10 mm trocar with a camera. Then, the patient was placed in the Trendelenburg position at approximately 20°, and the remaining trocars were inserted under visual control. Noteworthy configurations included the camera port, which was positioned in the umbilicus on the midline, with 5 mm robotic ports on the right and left mid-clavicular lines in alignment with the umbilicus. An assistant port of 5 mm was situated about 8 cm to the right of the umbilicus. The configuration of the trocar placement in a robot-assisted hysterectomy for non-oncological indications is presented in Fig. 2. In the case of a robot-assisted hysterectomy performed for oncological indications, a 10 mm suprapubic trocar was inserted, through which the removed lymph nodes were extracted in endo-bags (Fig. 3). In our robot-assisted surgeries, we most frequently used the following surgical instruments: monopolar hook, monopolar curved scissors, needle holder and bipolar Maryland grasper (Fig. 4). In addition to robotic tools, bipolar forceps and a suction device were prepared for the assistant. The following instruments were usually inserted through the trocars: left lower abdomen: bipolar Maryland grasper, right: monopolar scissors, right upper: assistant’s port (usually used to insert bipolar electrode or suction), umbilicus: used to insert a camera. Needle holders are usually inserted through the left and right torcars located in the lower abdomen. Before the operation began, a uterine manipulator, usually the SecuFix (Richard Wolf), was inserted into the uterus. The vagina was closed using robotic needle holders with a two-layer V-loc 2.0 suture. Removal of the lymph nodes in the iliac area was performed after removal of the uterus and closure of the vagina. Closing the vagina ended the robotic part of the procedure, after which the lymph nodes around the iliac vessels were removed laparoscopically.

All patients were qualified for surgical treatment on an outpatient basis and arrived at the hospital the day before surgery. After admission to the hospital, they underwent basic blood tests and a preoperative anaesthetic consultation. All patients fasted from the evening before surgery. On the day of the planned surgery, 500–1000 ml of crystalloids, were administered intravenously, and midazolam (7.5 mg) was administered orally for about 2 h before moving to the operating room. Approximately, 30 min before the start of the operation, the patient received a prophylactic dose of antibiotics (second-generation cephalosporins) intravenously. After the operation, the patient remained under close observation in the recovery room for about one hour. If, during this time, his vital parameters were stable and she did not require additional respiratory or circulatory support, she was transferred to the gynaecological ward for further observation. The patients remained in the observation room for up to 24 h, and during the stay, their vital parameters were monitored, including blood pressure, heart rate, oxygen saturation and diuresis.

On the morning of the first postoperative day, the catheter was removed from the bladder, and fluids were given to drink. In the afternoon, the patients received an easily digestible diet and were encouraged to get out of bed, gradually increasing their physical activity thereafter. On the first day after surgery, intravenous infusion fluids of 2000–2500 ml and painkillers were administered, and low-molecular-weight heparins were given in the evening.

### Data

Patient demographics and preoperative data included patient age, BMI, ASA, physical status, and surgical history. Intraoperative data consisted of operative time (skin incision to skin closure and console time in robotic procedures), complications during surgery, conversion to an alternative surgical technique (laparoscopic or open surgery), estimated blood loss during surgery, changes in blood morphology parameters, and requirements for blood transfusion. Postoperative outcome data included length of hospital stay (from procedure to discharge), complications following surgery within 90 days and their relatedness to the device, return to the operating room (OR) within 24 h of surgery, readmission to hospital within 30 days with reason, and any other relevant details (e.g. return to the OR) or mortality within 90 days of surgery. The evaluated endpoints of the study were surgical intraoperative outcomes (operative time, changes in blood morphology parameters, and conversion rate) and postoperative outcomes (duration of hospital postoperative stay, reoperation rate, overall complications rate and mortality rate). All postoperative complications were assessed according to the Clavien–Dindo classification. Histopathological examinations were conducted at the Department of Pathomorphology of the Regional Polyclinical Hospital in Grudziadz. The study was conducted with the approval of the Bioethics Committee at the Kujawsko-Pomorskie Regional Chamber of Physicians in Torun, under authorisation number 20/KB/2023.

### Statistical analysis

Statistical analyses were performed using the PQStat statistical package, version 1.8.4.152. The results of the quantitative scales between patient groups were compared using the Mann–Whitney *U* test. The normality of distribution was checked using the Shapiro–Wilk test and the homogeneity of rank variances using the Conover test. The results of the qualitative scales between patient groups were compared using the Chi-squared test and Fisher’s exact test. A probability level of *p* < 0.05 was considered significant, and a level of *p* < 0.01 was considered highly significant.

## Results

The study compared surgical intra- and postoperative outcomes of non-radical and radical hysterectomy performed laparoscopically and with the assistance of the Versius CMR surgical system (TLH vs TRH and RLH vs RRH, respectively). Patients included in each group did not differ in terms of: age, body mass index (BMI), American Society of Anaesthesiologists (ASA) category, comorbidities, or surgical history. The clinical characteristics of the study subjects are presented in Table 1.

**Table 1:** Clinical characteristics of study subjects

Age (years)	TLH	TRH	RLH	RRH
Mean	57.2	59.0	69.8	64
*p*	0.6353		0.0945	
BMI (kg/m^2^)				
Mean	31.3	27.8	32.5	33.9
*p*	0.1607		0.5259	
ASA				
I/II	14 (73.68%)	9 (64.29%)	6 (60%)	4 (57.14%)
III/IV	5 (26.32%)	5 (35.71%)	4 (40%)	3 (42.86%)
*p*	0.5615		0.9062	
Surgical history				
No	16 (84.21%)	13 (92.86%)	10 (100%)	6 (85.71%)
Yes	3 (15.79%)	1 (7.14%)	0 (0%)	1 (14.29%)
*p*	0.4519		0.2179	

*TLH* total laparoscopic hysterectomy, *TRH* total robotic hysterectomy, *RLH* radical laparoscopic hysterectomy, *RRH* radical robotic hysterectomy

Non-oncological diseases were indications for laparoscopic surgery in 18 cases (62.07%) and robotic surgery in 10 cases (47.62%), while oncological diseases were indications for laparoscopic surgery in 11 cases (37.93%) and robotic surgery in 11 cases (52.38%). No significant (*p* > 0.05) differences of the distribution of indications was found between the groups of patients undergoing laparoscopic and robotic procedures (Table 2).

**Table 2:** Oncological and non-oncological indications for surgery in groups of patients undergoing laparoscopic and robotic procedures

Indications	Procedure			
	Laparoscopic		Robotic	
	N	%	N	%
Oncological	11	37.93	11	52.38
Non-oncological	18	62.07	10	47.62
Cochran’s condition	Met			
Pearson’s Chi^2^ statistic	1.0321			
Degrees of freedom	1			
*p* value	0.3097			
Fisher’s exact test (*p* =)	0.3912			

**Fig. 5:**
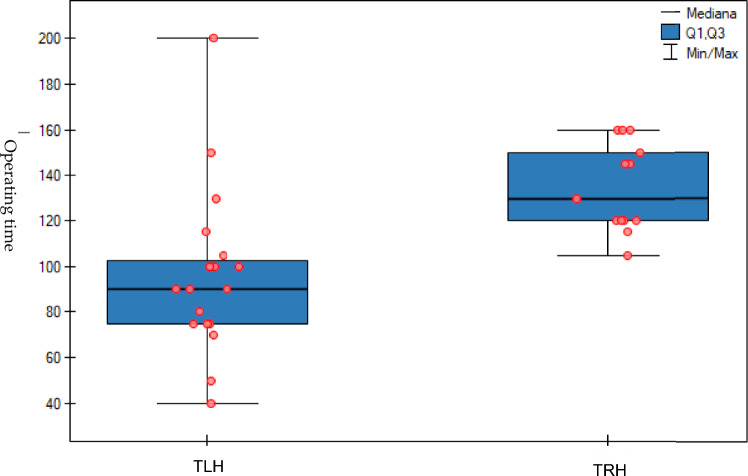
In case of total robotic assisted hysterectomy (TRH), operating time was significantly longer comparing to total laparoscopic hysterectomy (TLH)

Based on the statistical analyses, it was found that in the case of non-radical hysterectomy, the duration of surgery differed significantly (96.5 vs. 134.6 min, *p* < 0.01) between the groups of patients undergoing laparoscopic and robotic surgery. In the laparoscopic group, the range of results was more diverse than in the group in which surgery were performed using the robotic technique, and in this group, the duration of surgery was generally longer than in the laparoscopic group (Fig. 5). However, in the case of radical hysterectomy cases, the duration of the operation did not differ significantly (157 vs. 183 min, *p* > 0.05) between the groups of patients undergoing laparoscopic and robotic surgeries. We also analysed console times for individual operators. In June 2023, the console time of the first operator was 106 min, and that of the second operator was 111 min. These times were reduced to 71 and 72 min respectively, by December 2023. There were also no statistically significant differences in blood parameters after the laparoscopic and robotic surgeries. Postoperative haemoglobin, haematocrit, and erythrocyte levels were compared between both groups, with no statistically significant differences found. None of the operated patients required transfusion of packed red blood cells or any other blood product in the postoperative period. After receiving the robotic and laparoscopic surgeries, patients were discharged an average of 2.31 vs. 2.23 days later, respectively (*p* > 0.05), in which regard the two compared groups did not differ significantly (Tables 3, 4). In both radical and non-radical hysterectomies, there was no need to convert to another type of surgical treatment. In no case was it necessary to return to the OR within 24 h of surgery or to be readmitted to the hospital within 30 days. There was also no death reported within 90 days after surgery in any of the study groups. In both groups, in the cases of non-radical and radical hysterectomy, there were similar, non-statistically significant rates of complications. In the group of patients undergoing robotic hysterectomy, these were mainly Class I complications, according to the Clavien–Dindo scale (Table 5). All patients are provided with outpatient care at our centre, undergo regular check-ups and after 6 months after the surgery they have imaging tests performed. During the follow-up period, we have not recorded a case of recurrence after our oncological treatment.

**Table 3:** Results after non-radical hysterectomy

Non-radical hysterectomy											
		Operating time (min)		ΔHGB (g/dl)		ΔHCT (%)		ΔRBC (10^6/µL)		Discharged days	
		TLH *N* = 19	TRH *N* = 14	TLH *N* = 19	TRH *N* = 14	TLH *N* = 19	TRH *N* = 14	TLH *N* = 19	TRH *N* = 14	TLH *N* = 19	TRH *N* = 14
Mean		96.5	134.6	1.1	1.6	3.5	5.0	0.4	0.5	2.3	2.2
Standard deviation		35.7	19.4	0.7	0.5	2.5	1.8	0.2	0.1	0.8	0.4
Median		90	130	1.1	1.7	3.4	5.4	0.4	0.5	2	2
Min		40	105	−0.1	0.6	0	2.3	−0.1	0.2	2	2
Max		200	160	2.4	2.4	7.9	7.6	0.8	0.8	5	3
Q1		75	120	0.7	1.2	1.25	3.7	0.2	0.5	2	2
Q3		102.5	150	1.7	1.9	5.5	6.4	0.6	0.6	2	2
Mann–Whitney* U* test	Z	3.542		1.6543		1.5928		1.5926		0.3105	
	P	0.0004		0.0981		0.1112		0.1112		0.7562	

*TLH* total laparoscopic hysterectomy, *TRH* total robotic hysterectomy, *ΔHGB* postoperative loss of haemoglobin, *ΔHCT* postoperative loss of haematocrit, *ΔRBC* postoperative loss of red blood cells, *Min* minimum, *Max* maximum, *Q1* first quartile, *Q3* third quartile

**Table 4:** Results after radical hysterectomy

Radical hysterectomy											
		Operating time (min)		ΔHGB (g/dl)		ΔHCT (%)		ΔRBC (10^6/µL)		Discharged days	
		RLH N = 10	RRH *N* = 7	RLH *N* = 10	RRH *N* = 7	RLH *N* = 10	RRH *N* = 7	RLH *N* = 10	RRH *N* = 7	RLH *N* = 10	RRH *N* = 7
Mean		157	183.1	2.16	1.6	6.4	4.7	0.7	0.5	3	2.6
Standard deviation		32.5	55.6	0.8	0.4	3.1	1.9	0.2	0.2	1.4	0.5
Median		147.5	172.5	2	1.5	5.8	4.0	0.7	0.5	2.5	3
Min		115	115	0.8	1	1.4	1.7	0.2	0.2	2	2
Max		215	305	3.6	2.5	11.2	8.1	1.1	0.8	6	3
Q1		141.2	157.5	1.9	1.2	5.3	3.9	0.6	0.4	2	2
Q3		175	192.5	2.6	1.8	8.2	5.9	0.6	0.6	3	3
Mann–Whitney* U* test	Z	1.1135		1.4691		1.0229		1.7335		−0.0489	
	P	0.2655		0.1418		0.3064		0.083		0.961	

*RLH* radical laparoscopic hysterectomy, *RRH* radical robotic hysterectomy, *Δ HGB* postoperative loss of haemoglobin, *Δ HCT* postoperative loss of haematocrit, *Δ RBC* postoperative loss of red blood cells, *Min* minimum, *Max* maximum, *Q1* first quartile, *Q3* third quartile

**Table 5:** Postoperative complications according to Clavien–Dindo classification

Patient no	Indications	Type of surgery	Complications	Days after the surgery	Clavien–Dindo classification
1	Endometrioid cancer	RLH + pelvic lymphadenectomy	Vaginal cuff partial dehiscence	17	II
2	Cervical dysplasia	TLH + BSO	Vaginal cuff infection	16	II
3	Endometrial hyperplasia	TLH + BSO	Abdominal wound dehiscence	5	IIIA
4	Endometrial hyperplasia	TRH + BSO	Discomfort, femoral pain	5	I
5	Endometrial hyperplasia	TRH + BSO	Discomfort, fever	7	I

*RLH* radical laparoscopic hysterectomy, *TLH* total laparoscopic hysterectomy, *BSO* bilateral salpingo-oophorectomy, *TRH* total robotic hysterectomy

## Discussion

Extensive research has underscored the numerous advantages of MIS compared to traditional open surgery, including diminished blood loss, reduced intraoperative and postoperative complications, shorter hospital stays, and improved clinical outcomes [8]. Despite these benefits, MIS comes with challenges, such as restricted instrument movement and two-dimensional vision, which pose difficulties for precise dissection and suturing. Proficiency in MIS may require an extended learning period, and discomfort has been reported due to laparoscopic instrument design. Such limitations include non-ergonomic instrument design, restricted reach, limited haptic feedback, and the absence of depth perception. Robot-assisted MIS has made significant strides in addressing these challenges by offering a stable, magnified, three-dimensional view, as well as tremor filtration, motion scaling, and articulated or wristed instruments with enhanced movement capabilities. These advancements have enhanced surgeons’ visualisation, dexterity, and precision, thereby allowing for the limitations associated with conventional laparoscopy to be overcome. Furthermore, the potential of robot-assisted surgery to ease such technical challenges may also minimise the learning curve, thereby making MIS more accessible to a diverse patient population, including those with a high BMI [9].

Based on the statistical analyses of the gathered material, it was observed that in the case of non-radical hysterectomy, the duration of surgery was significantly different between the laparoscopic and robotic surgery groups. The results in the robot group were higher than in the laparoscopic group. However, for radical hysterectomy, the duration of the operation did not differ significantly between the laparoscopic and robotic surgery groups. Considering that the operating team had extensive experience in performing minimally invasive laparoscopic hysterectomies, the longer duration of non-oncologic surgeries could be attributed to the fact that the operators initially introduced robotic procedures in non-oncologic patients. Therefore, the first robot-assisted surgeries involved a cautious and time-consuming introduction and acquired mastery of the new surgical technique, which extended the duration of the operations. In addition, specific procedures related to robot assistance during surgery require a time-consuming setup process and preparation of the BSUs and robotic tools. In contrast, oncologic surgeries were performed after a certain period of acclimatisation to the robot’s operation, and therefore, the duration of the operations for oncologic patients did not differ significantly between the laparoscopic and robotic groups. In the case of non-radical hysterectomies performed for benign lesions, the median times from incision to skin closure obtained in our study were shorter compared to those obtained by Borse et al. [10].

The statistical analysis of our collected data did not reveal significant differences in terms of postoperative loss of haemoglobin, haematocrit, and erythrocyte levels between the group of patients operated on with laparoscopic and robotic techniques. In addition, regarding the average length of postoperative hospitalisations, there were no significant differences between the groups. Moreover, in the case of non-radical hysterectomies performed laparoscopically and robotically, there were no statistically significant differences in the percentages of cases converted to another type of surgery. In the case of radical hysterectomy, there was no need to convert to another type of surgery in either group. The results thus point to the feasibility, safety and effectiveness of this approach, which was also demonstrated by Puntambekar et al. [11].

The physical challenges faced by surgeons during MIS, such as muscular strains and fatigue, can be intensified by the demanding nature of gynaecological procedures, wherein surgeons must operate in a parallel axis to the pelvis. Neck and back pain, which are frequently documented occupational hazards, leads to a significant number of sick days and early retirements among surgeons [12]. Evidence supports the idea that surgical robots offering the option for surgeons to be seated with armrests while operating can alleviate muscular strain on the legs, shoulders, and back, thereby reducing surgeon energy consumption during simulated MIS. The utilisation of this approach not only addresses physical burdens, but also contributes to a more sustainable and enduring surgical career [13]. One of the features of the Versius CMR system that distinguishes it from the da Vinci system is the ability of the operator to assume a standing position behind the console. The position of the BSUs around the table, despite their easy removal, may not allow the assistant surgeon to adopt a proper position. This can be uncomfortable when performing high-risk gestures, such as clipping or stapling. In our study, general fatigue and postural discomfort during robot-assisted surgery were described as acceptable by both operators. Undoubtedly, a more detailed assessment will follow after more robot-assisted surgical procedures are performed.

The master console, operated by hand, ensures a three-dimensional view with the use of passive polarised glasses. Its components include a handle with a lever for instrument jaw opening, a clutch button, an energy activation button, a joystick for endoscope control, and an energy LED indicator. One joystick manages the camera rotation and distance, while the other controls the movements of the camera arm. The BSUs, each with a limited weight of 100 kg and manually positioned, must be covered by a sterile drape before platform docking. Upon reaching the desired position, a brake is activated to stabilise the BSU on the floor. A 12 mm endoscope and 5 mm instruments can then be mounted and rotated in a cone-like fashion, thereby completing the process of ‘port training’. No specific trocars are required, but balloon-cuffed ones are recommended to prevent displacement, and specific insufflators or energy devices are not necessary. The Versius surgical system integrates a surgeon console, instrument BSU (allowing for two or three units based on procedure and surgeon preference, with three units utilised in this study), and a visualisation BSU. In utilising energy instruments, such as the monopolar hook and bipolar Maryland grasper, the system allows for flexible port placement, as evidenced in a study detailing common port positions and OR layouts. Portable BSUs offer flexibility in port placement, thereby allowing for adherence to surgeons’ preferences from conventional MIS. An important fact worth emphasising that results from the location of the trocars in a similar fashion to the traditional, laparoscopic methods of MIS is the possibility of a smooth transition from robotic to laparoscopic surgery if unexpected complications arise. This usually does not require the introduction of additional trocars, but only the removal of robotic tools and the movement of BSU units away from the operating table. The Versius robot’s most versatile feature is the independence of its robotic arms; however, this characteristic also presents a notable limitation. The potential for clashes among the arms can result in instrument blockage, leading to longer operative times. The company has provided some clinical suggestions to mitigate this inconvenience. As highlighted by other authors, achieving an optimal position requires calibration based on the patient’s body habitus and proper training in cadaveric models [14]. This approach differs from the current robotic standard, which typically involves linear trocar placement, rapid docking, and minimal interference among instruments [13]. To prevent clashes, continuous monitoring of the robotic arms’ movements is essential. In many instances, a fourth robotic arm is employed to apply countertraction and is subsequently removed to minimise conflicts with the three other active arms. In robot-assisted surgeries performed by our team, we usually used two instrument BSUs, and one endoscopic BSU. Clashes occurred during the first operations and resulted mainly from suboptimal positioning of the BSU in relation to the operating table, patient, and instrumentation. As we gained more experience in positioning the BSUs, interruptions due to clashes occurred less frequently. It should also be noted that the Versius surgical robot by CMR surgical, unlike the daVinci system, is characterised by an open console. The use of such a solution, according to some authors, allows for better communication with other members of the surgical team [15].

The basic condition for the success of the operation is a thorough assessment and appropriate qualification for treatment; this is particularly important in the case of implementing a new, minimally invasive surgical technique using a robotic system. Such an assessment primarily involves a detailed interview regarding past surgical treatments, gynaecological examinations and ultrasonographic assessments. A gynaecological examination is crucial for evaluating the size and mobility of the uterus and adnexa, thereby enabling an assessment of the potential use of a uterine manipulator and the extraction of the excised uterus through the vaginal route. Another vital qualifying factor is the performance of a dynamic ultrasound examination, which can confirm the mobility of pelvic organs in relation to the bladder, rectum, and parietal peritoneum. Finally, ensuring the safe execution of surgical procedures for patients with multiple comorbidities requires attentive and dedicated care from the anaesthesiologist throughout the operation [16].

The analysis of complications in our collected material allowed us to conclude that the incidence rates in this regard were similar between the laparoscopic and robotic groups. In the group undergoing laparoscopic surgeries, complications occurred in three cases, which were mainly classified as wound dehiscence or postoperative wound infection, which fell into Categories II and III of complications according to the Clavien–Dindo scale [17]. In the case of patients receiving robotic surgeries, complications were noted in two cases, which were classified as Category I complications according to Clavien–Dindo and primarily related to the occurrence of discomfort and pain in the operated area. In all cases, interventions were implemented, leading to the resolution of symptoms within a few days. Our results in this sense were consistent with those, obtained by Bruno et al., who compared complications after hysterectomy performed laparoscopically and using the da Vinci robot. In this study, the percentage of complications after robotic surgery was lower compared to those associated with laparoscopy, but in our research, there were no severe complications requiring reoperation [18].

Over the past decade, several robotic surgical systems have emerged in addition to da Vinci, some of which have achieved clinical approval, although none have yet achieved global availability. When comparing the first generation of the Versius system, it is important to note that we are contrasting it with the fourth generation of the da Vinci system. The main distinguishing features between the two systems are modularity and the open console of the Versius system. Undoubtedly, a significant advantage of the daVinci system is the ability to utilise indocyanine green and well-established, precise tools. However, the daVinci system faces frequent criticisms, including challenges in communication between the surgeon and the surgical team due to the closed console system, lack of haptic feedback, rigidity of arm placement, cost and its large size [19]. The Versius robotic system by CMR stands out with its open console design, which enhances communication among the surgical team, allows direct observation of the robot’s arm positions, and offers the surgeon the flexibility to sit or stand. Similar open console solutions are featured in the Senhance and Hugo RAS surgical robots [20]. The Senhance robotic platform offers several advantages over the da Vinci system. It uses a multiport configuration accommodating up to four independent robotic arms on separate carts, eliminating the need for large dedicated operating theatres and making it compatible with most existing ones. The surgeon operates from an ergonomically designed open console with a monitor providing 3D high-definition visualisation through polarised glasses. The infrared eye-tracking system, known as eye-sensing control technology, simplifies camera manipulation by responding to the surgeon’s eye movements. In addition, standard laparoscopic trocars are used for introducing robotic instruments, allowing quick conversion to conventional laparoscopy in emergencies. The system also autonomously calculates the force exerted by the robotic arms on the trocars, preventing excessive traction on insertion points. One of Senhance’s most significant advantages is its haptic feedback, which aids in delicate tissue handling and intracorporeal suturing [21]. The Hugo RAS system features a console with two arm controllers operated with a pistol-like grip, and a footswitch that controls the camera, energy source, and reserve arm. It includes four separate arm carts, each with six joints to enhance the range of motion, and uses 3D glasses for head tracking technology [22]. Although the da Vinci system has dominated robotic surgery, emerging systems offer unique features such as open consoles, modular designs, compatibility with traditional instruments, reduced size, and lower costs. The growing competition and diversity in the field provide alternative options with various benefits over the da Vinci system.

The main strengths of our study were: a homogeneous group of oncological patients took part in the study, all procedures were performed by the same level of gynaecologic oncologists with the same technique, and uniform postoperative procedures were used in both groups of patients. One of the main limitations of this paper is the small number of cases of robotic surgery included in the study. This was mainly due to the lack of daily availability of the Versius robot in the centre where the authors worked. Other limitations of this publication are: the high heterogeneity of non-oncological diagnoses that constituted indications for surgery in relation to the size of the group and the retrospective character of the study. Nevertheless, due to the encouraging results of this study, there is reason to conduct additional studies to confirm their replicability in a larger group of operated patients.

In summary, based on the analysis of the data we collected, the lack of differences in loss of blood parameters, complications, mortality rate and postoperative hospitalisation times, minimally invasive surgery performed with the Versius CMR surgical system appears to be a safe and effective alternative to laparoscopic surgery in gynaecology.

## Conclusions

Based on surgical intraoperative outcomes, hysterectomy with the assistance of the Versius CMR surgical system is comparable to the procedure performed laparoscopically.

No differences observed in the postoperative course of patients operated on with the Versius CMR surgical system suggest that it may constitute a secure and efficient substitute for laparoscopic surgery.

## Data Availability

The data used to support the findings of this study are available from the corresponding author upon request.

## Abbreviations

MIS: Minimally invasive surgery
BMI: Body mass index
ASA: American Society of Anaesthesiologists
TLH: Total laparoscopic hysterectomy
BSO: Bilateral salpingo-oophorectomy
BS: Bilateral salpingectomy
TRH: Total robotic assisted hysterectomy
RLH: Radical laparoscopic hysterectomy
RRH: Radical robotic assisted hysterectomy
BSU: Bedside unit
OR: Operating room

## References

[1] Diaz-Arrastia C, Jurnalov C, Gomez G, Townsend C Jr (2002) Laparoscopic hysterectomy using a computer-enhanced surgical robot. Surg Endosc 16(9):1271–1273. 10.1007/s00464-002-8523-5 12085153 10.1007/s00464-002-8523-5

[2] Jernigan AM, Auer M, Fader AN, Escobar PF (2012) Minimally invasive surgery in gynecologic oncology: a review of modalities and the literature. Womens Health (Lond) 8(3):239–250. 10.2217/whe.12.13 22554172 10.2217/whe.12.13

[3] Palep JH (2009) Robotic assisted minimally invasive surgery. J Minim Access Surg 5(1):1–7. 10.4103/0972-9941.51313 19547687 10.4103/0972-9941.51313 PMC2699074

[4] Leal Ghezzi T, Campos CO (2016) 30 Years of robotic surgery. World J Surg 40(10):2550–2557. 10.1007/s00268-016-3543-9 27177648 10.1007/s00268-016-3543-9

[5] Eoh KJ, Kim TJ, Park JY, Kim HS, Paek J, Kim YT (2023) Robot-assisted versus conventional laparoscopic surgery for endometrial cancer: long-term comparison of outcomes. Front Oncol 15(13):1219371. 10.3389/fonc.2023.1219371 10.3389/fonc.2023.1219371 PMC10540847 37781200

[6] Brassetti A, Ragusa A, Tedesco F, Prata F, Cacciatore L, Iannuzzi A, Bove AM, Anceschi U, Proietti F, D’Annunzio S, Flammia RS, Chiacchio G, Ferriero M, Guaglianone S, Mastroianni R, Misuraca L, Tuderti G, Simone G (2023) Robotic surgery in urology: history from PROBOT® to HUGO™. Sensors (Basel) 23(16):7104. 10.3390/s23167104 37631641 10.3390/s23167104 PMC10458477

[7] Soumpasis I, Nashef S, Dunning J, Moran P, Slack M (2023) Safe implementation of a next-generation surgical robot: first analysis of 2083 cases in the Versius surgical registry. Ann Surg 278(4):e903–e910. 10.1097/SLA.0000000000005871 37036097 10.1097/SLA.0000000000005871 PMC10481922

[8] Pickett CM, Seeratan DD, Mol BWJ, Nieboer TE, Johnson N, Bonestroo T, Aarts JW (2023) Surgical approach to hysterectomy for benign gynaecological disease. Cochrane Database Syst Rev 8(8):CD003677. 10.1002/14651858.CD003677.pub6 37642285 10.1002/14651858.CD003677.pub6 PMC10464658

[9] Haig F, Medeiros ACB, Chitty K, Slack M (2020) Usability assessment of Versius, a new robot-assisted surgical device for use in minimal access surgery. BMJ Surg Interv Health Technol 2(1):e000028. 10.1136/bmjsit-2019-000028 35047788 10.1136/bmjsit-2019-000028 PMC8749256

[10] Borse M, Godbole G, Kelkar D, Bahulikar M, Dinneen E, Slack M (2022) Early evaluation of a next-generation surgical system in robot-assisted total laparoscopic hysterectomy: a prospective clinical cohort study. Acta Obstet Gynecol Scand 101(9):978–986. 10.1111/aogs.14407 35861102 10.1111/aogs.14407 PMC9564672

[11] Puntambekar SP, Goel A, Chandak S, Chitale M, Hivre M, Chahal H, Rajesh KN, Manerikar K (2021) Feasibility of robotic radical hysterectomy (RRH) with a new robotic system. Experience at galaxy care laparoscopy institute. J Robot Surg 15(3):451–456. 10.1007/s11701-020-01127-x 32710253 10.1007/s11701-020-01127-x

[12] Stucky CH, Cromwell KD, Voss RK, Chiang YJ, Woodman K, Lee JE, Cormier JN (2018) Surgeon symptoms, strain, and selections: systematic review and meta-analysis of surgical ergonomics. Ann Med Surg (Lond) 9(27):1–8. 10.1016/j.amsu.2017.12.013 10.1016/j.amsu.2017.12.013 PMC5832650 29511535

[13] Huscher C, Marchegiani F, Cobellis F, Tejedor P, Pastor C, Lazzarin G, Wheeler J, Di Saverio S (2022) Robotic oncologic colorectal surgery with a new robotic platform (CMR Versius): hope or hype? A preliminary experience from a full-robotic case-series. Tech Coloproctol 26(9):745–753. 10.1007/s10151-022-02626-9 35637355 10.1007/s10151-022-02626-9 PMC9360145

[14] Kelkar D, Borse MA, Godbole GP, Kurlekar U, Slack M (2021) Interim safety analysis of the first-in-human clinical trial of the Versius surgical system, a new robot-assisted device for use in minimal access surgery. Surg Endosc 35(9):5193–5202. 10.1007/s00464-020-08014-4 32989548 10.1007/s00464-020-08014-4 PMC8346419

[15] Brownlee EM, Slack M (2022) The role of the versius surgical robotic system in the paediatric population. Children (Basel) 9(6):805. 10.3390/children9060805 35740742 10.3390/children9060805 PMC9222178

[16] Suryawanshi CM, Shah B, Khanna S, Ghodki P, Bhati K, Ashok KV (2023) Anaesthetic management of robot-assisted laparoscopic surgery. Indian J Anaesth 67(1):117–122. 10.4103/ija.ija_966_22 36970478 10.4103/ija.ija_966_22 PMC10034944

[17] Dindo D, Demartines N, Clavien PA (2004) Classification of surgical complications: a new proposal with evaluation in a cohort of 6336 patients and results of a survey. Ann Surg 240(2):205–213. 10.1097/01.sla.0000133083.54934.ae 15273542 10.1097/01.sla.0000133083.54934.ae PMC1360123

[18] Bruno M, Legge F, Gentile C, Carone V, Stabile G, Di Leo F, Ludovisi M, Di Florio C, Guido M (2022) Risk assessment model for complications in minimally invasive hysterectomy: a pilot study. Int J Environ Res Public Health 20(1):234. 10.3390/ijerph20010234 36612556 10.3390/ijerph20010234 PMC9819802

[19] Namdarian B, Dasgupta P (2018) What robot for tomorrow and what improvement can we expect? Curr Opin Urol 28(2):143–152. 10.1097/MOU.0000000000000474 29303916 10.1097/MOU.0000000000000474

[20] Salkowski M, Checcucci E, Chow AK, Rogers CC, Adbollah F, Liatsikos E, Dasgupta P, Guimaraes GC, Rassweiler J, Mottrie A, Breda A, Crivellaro S, Kaouk J, Porpiglia F, Autorino R (2023) New multiport robotic surgical systems: a comprehensive literature review of clinical outcomes in urology. Ther Adv Urol 5(15):17562872231177780. 10.1177/17562872231177781 10.1177/17562872231177781 PMC10265325 37325289

[21] McCarus SD (2021) Senhance robotic platform system for gynecological surgery. JSLS 25(1):00075. 10.4293/JSLS.2020.00075 10.4293/JSLS.2020.00075 PMC8035830 33880002

[22] Prata F, Ragusa A, Tempesta C, Iannuzzi A, Tedesco F, Cacciatore L, Raso G, Civitella A, Tuzzolo P, Callè P, Pira M, Pino M, Ricci M, Fantozzi M, Prata SM, Anceschi U, Simone G, Scarpa RM, Papalia R (2023) State of the art in robotic surgery with hugo RAS system: feasibility, safety and clinical applications. J Pers Med 13(8):1233. 10.3390/jpm13081233 37623483 10.3390/jpm13081233 PMC10456103

